# Supplementary figures and images for: Spike substitution T813S increases Sarbecovirus fusogenicity by enhancing the usage of TMPRSS2
Source: PLoS Pathog. 2023 May 17;19(5):e1011123. doi: 10.1371/journal.ppat.1011123 (PMC10228797; doi:10.1371/journal.ppat.1011123)

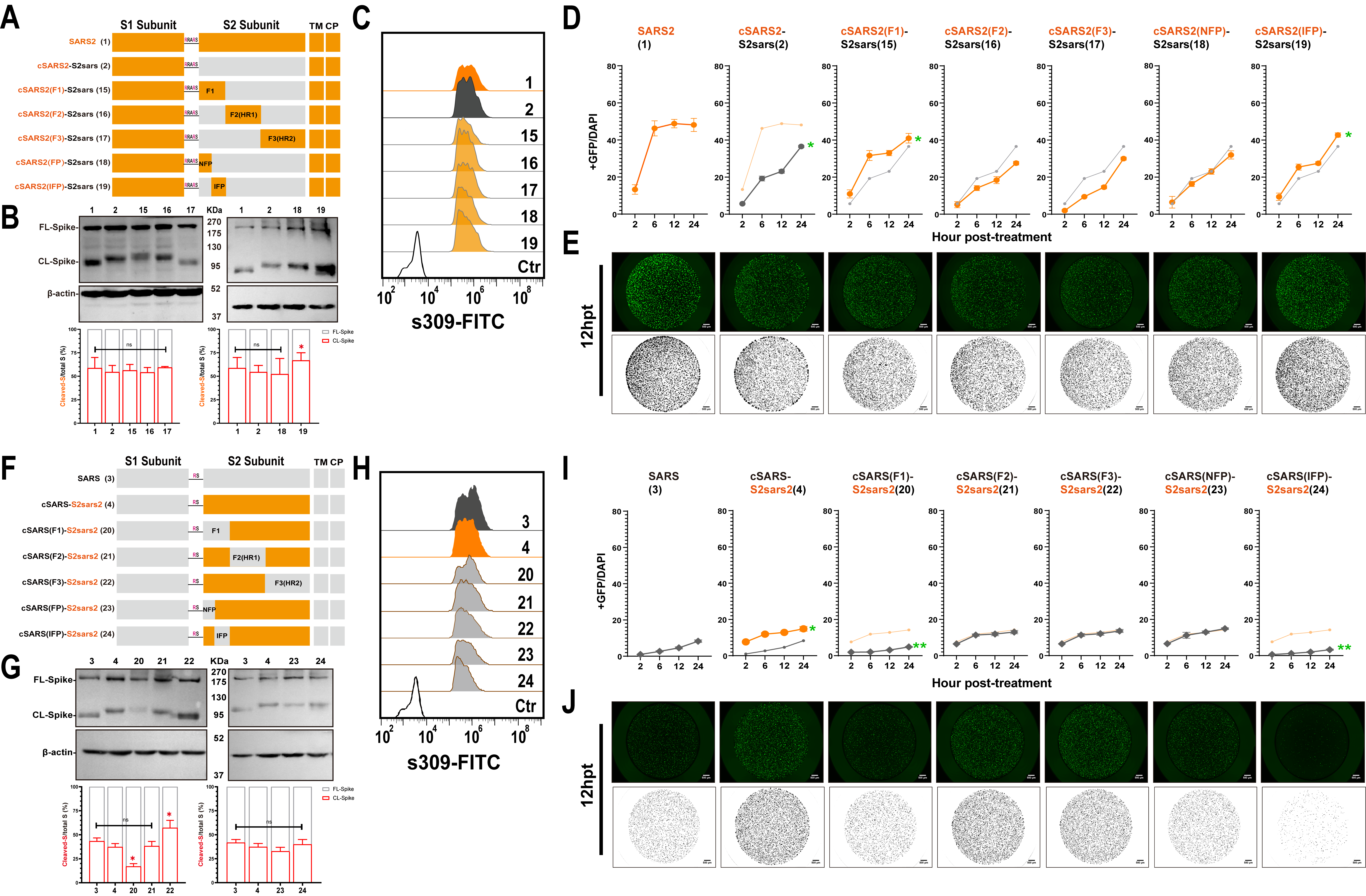

Supplement: S1 Fig — (A and F) Schematic diagram of the S2 motif chimeric Spike. The S2 was divided into 3 parts as Fig 2 and to replace the corresponding area in turn. The F1 was further divided into two parts just as Fig 2 did. The numbers in parentheses are identical to those in Fig 3B–3E and 3F–3J. (B and G) Western blot. A representative blot of S-expressing cells (top) and quantified band intensity (the ratio of CL-S to the FL-S plus CL-S proteins) (bottom) are shown. (C and H) Flow cytometry. The summarized results of the surface S expression were shown. s309 antibody and mouse anti-human IgG-FITC were used respectively. (D and I) Spike-based fusion assay. The fusion activity was quantified by measuring the ratio of GFP+ area to DAPI area by imaging at different times (2, 6, 12 and 24hpt). The results for SARS-CoV-2, Spike4, 15–19 or SARS-CoV, Spike2, 10–14 were shown as Saffron and grey lines, respectively. (E and J) Representative images of cell-cell fusion. Scale bar: 500 μm. Results are means +/- SD from at least three fields per condition. Results are representative of at least three independent experiments. In B and G, statistically significant differences between parental S (Spike2 or Spike4) and chimeric Spikes were determined by a two-sided paired t test (*: p<0.05). In D and I, statistically significant differences between parental S (Spike2 or Spike4) and chimeric Spikes were determined by Student’s test at each point (*: p<0.05, **: p<0.01). (TIF) [file ppat.1011123.s001.tif]

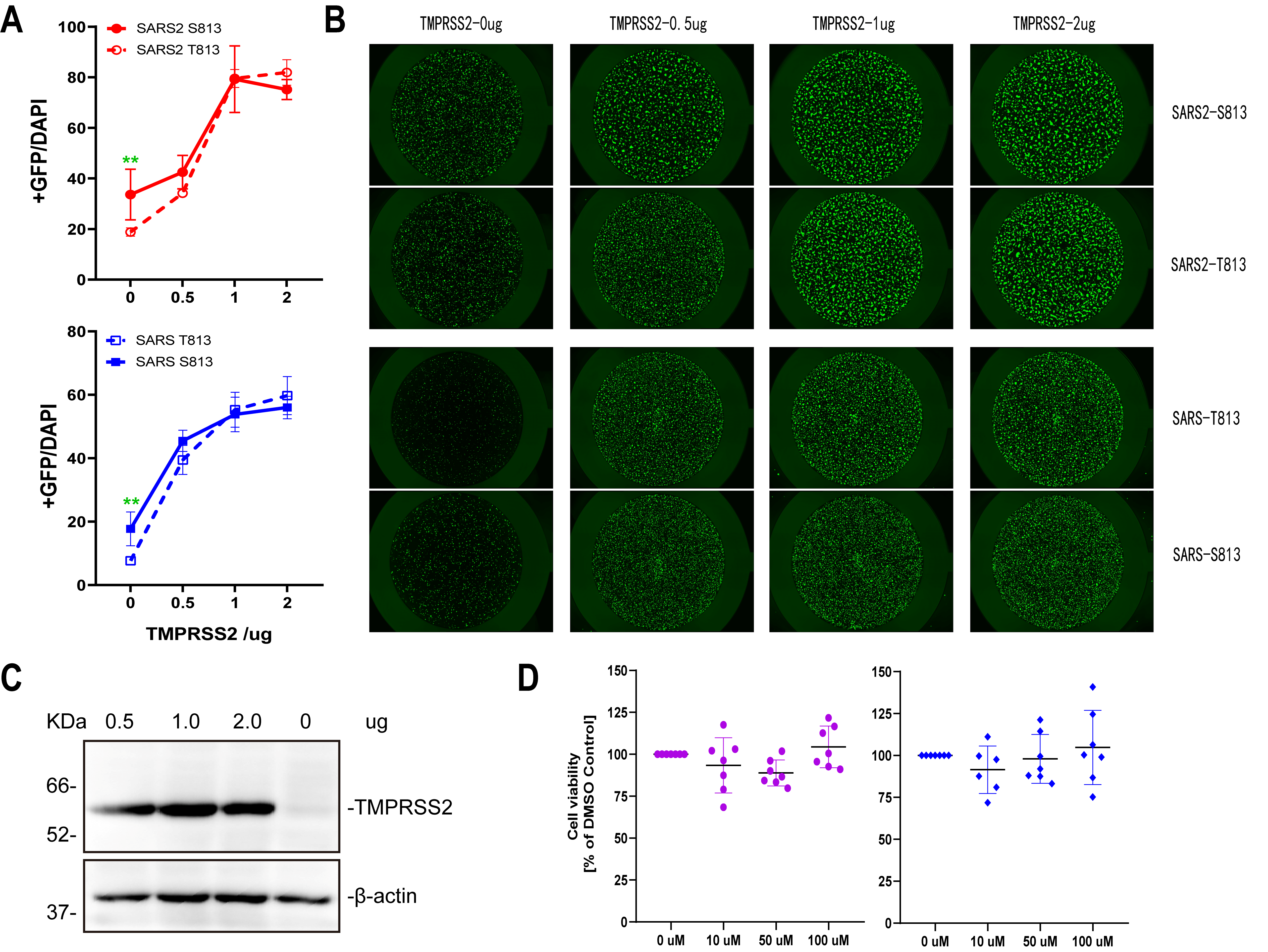

Supplement: S2 Fig — (A) Spike-based fusion assay. The fusion activity was quantified by measuring the ratio of GFP+ area to DAPI area by imaging at different concentration of TMPRSS2 (0, 0.5, 1 and 2 μg). The results for SARS-CoV-2 and SARS-CoV were shown as Red and grey lines, respectively. (B) Representative images of cell-cell fusion. Scale bar: 500 μm. (C) Western blot. A representative blot of 293T cell lysates expressing TMPRSS2 with various concentrations. Beta-actin was used as a control. (D) Cell viability assay. The cell viability with different doses of Camostat and E64d was evaluated by CCK8 assay. Results are means +/- SD from at least three fields per condition. Results are representative of at least three independent experiments. Statistically significant differences (**: p<0.01) between S813 Spike and T813 Spike were determined by Student’s test at each point (C, F and G). (TIF) [file ppat.1011123.s002.tif]

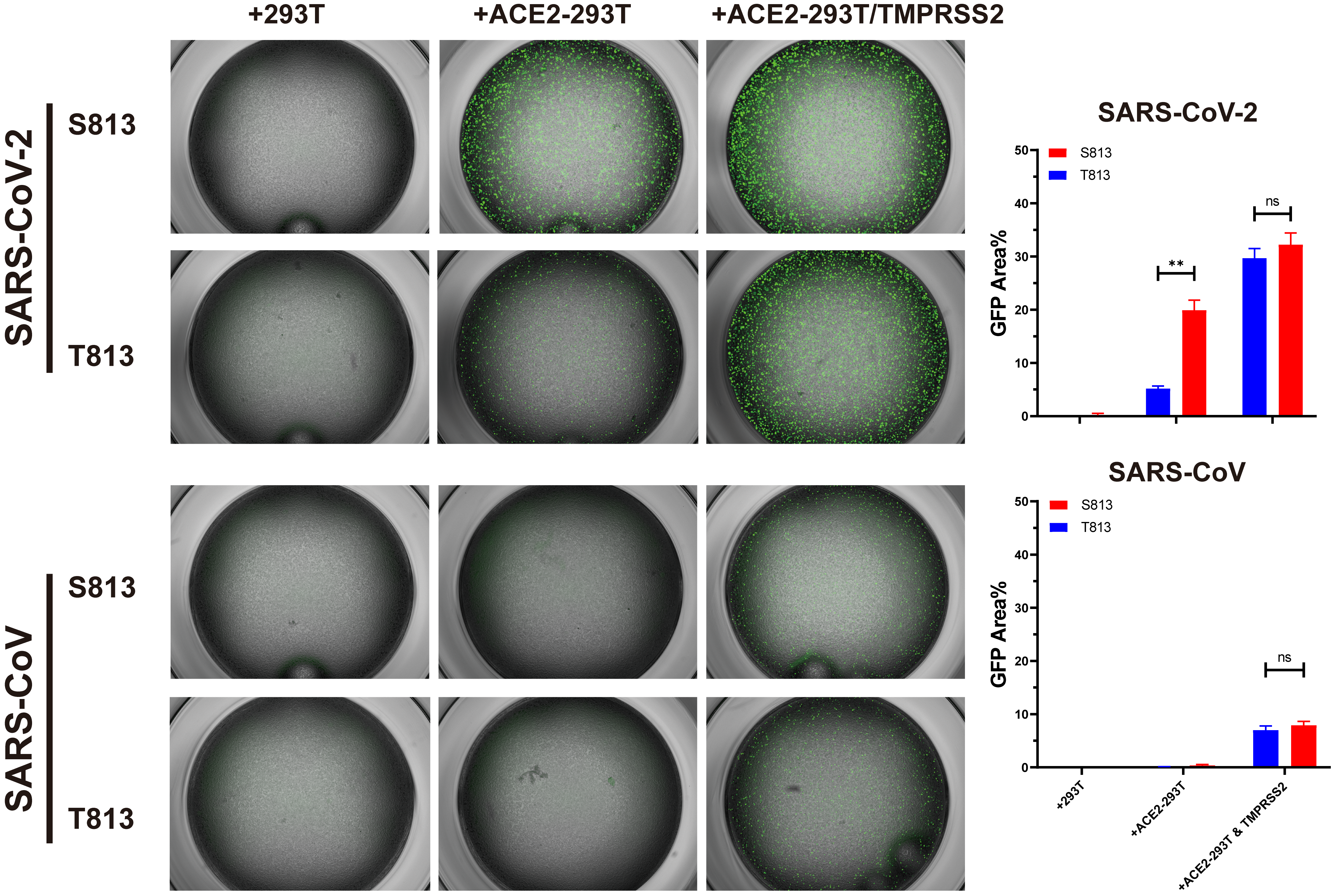

Supplement: S3 Fig — The fusion activity of Spikes with S813 or T813 were quantified in non-trypsin condition, by measuring the ratio of GFP+ area to DAPI area at different concentration: Spike expressing cells mixing with 293T cells, ACE2-293T cells or ACE2-293T/TMPRSS2 cells respectively. The results for S813 S and T813 S were shown as Red and Blue histogram, and the disturbance of fusion by S813T mutation is still observed in SARS-CoV-2, but not in SARS-CoV. After overexpressing TMPRSS2, the fusion ability increased both in SARS-CoV and SARS-CoV-2, and the influence of S813T mutation was disappeared. Representative cell-cell fusion images of SARS-CoV-2 (up) and SARS-CoV (down) were shown. Scale bar: 500 μm. Results are means +/- SD from at least three fields per condition. Results are representative of at least three independent experiments. Statistically significant differences (**: p<0.01) between S813 Spike and T813 Spike were determined by Student’s test at each point. (TIF) [file ppat.1011123.s003.tif]
